# Targeting *Plasmodium falciparum* Schizont Egress Antigen-1 in Infected Red Blood Cells: Docking-Based Fingerprinting, Density Functional Theory, Molecular Dynamics Simulations, and Binding Free Energy Analysis

**DOI:** 10.3390/ph18020237

**Published:** 2025-02-10

**Authors:** Hassan H. Almasoudi, Mohammed H. Nahari

**Affiliations:** Department of Clinical Laboratory Sciences, College of Applied Medical Sciences, Najran University, Najran 66462, Saudi Arabia; hhalmasoudi@nu.edu.sa

**Keywords:** PfSEA-1, structural modelling, docking, pharmacokinetics, MD simulation, MMGBSA

## Abstract

**Background:** Malaria remains a global health crisis, with the World Health Organization (WHO) reporting 241 million cases and 627,000 deaths worldwide in 2020, predominantly affecting Sub-Saharan Africa. The region accounted for 95% of cases and 96% of deaths, reflecting the immense challenges in malaria prevention and treatment. *Plasmodium falciparum* Schizont Egress Antigen-1 (PfSEA-1) is crucial in facilitating immune evasion and promoting the sequestration of infected red blood cells (RBCs), contributing to severe malaria symptoms, including cerebral malaria, and necessitates the urgent identification of novel or repurposed drugs targeting PfSEA1. **Methods:** The protein structure of PfSEA-1 (UniProt ID: A0A143ZXM2) was modelled in three dimensions, prepared, and subjected to a 50 ns molecular dynamics (MD) simulation to achieve a stable structure. The equilibrated structure was minimised for molecular docking against the DrugBank compound library. Docking analysis identified potential inhibitors, including Alparabinos, Dihycid, Ambenzyne, Amiflupipquamine, Ametchomine, and Chlobenethyzenol, with docking scores ranging from −8.107 to −4.481 kcal/mol. Advanced analyses such as interaction fingerprints, density functional theory (DFT), and pharmacokinetics evaluations were conducted. Finally, a 100 ns MD simulation in the NPT ensemble was performed to assess the stability of protein–ligand complexes, with binding free energy and total energy calculations derived from the simulation trajectories. **Results and Discussion:** The identified compounds exhibited satisfactory pharmacokinetic profiles and binding interactions with PfSEA-1. The MD simulations demonstrated overall stability, with minor fluctuations in some instances. Key intermolecular interactions were observed, supporting the binding stability of the identified compounds. Binding free energy calculations confirmed favourable interactions, underscoring their potential as therapeutic agents against Plasmodium falciparum. While the in silico results are promising, experimental validation is essential to confirm their efficacy and safety for clinical use. **Conclusion:** These findings highlight PfSEA-1 as a promising antimalarial target and identify potential inhibitors with strong binding affinities and favourable pharmacokinetics. While the computational results are encouraging, further in vitro and in vivo validation is necessary to confirm their therapeutic potential and facilitate future drug development.

## 1. Introduction

Malaria is a life-threatening disease caused by Plasmodium parasites, transmitted to humans through the bites of infected female Anopheles mosquitoes. Five species of Plasmodium can cause malaria in humans, with *Plasmodium falciparum* and *Plasmodium vivax* being the most prevalent. *P. falciparum* is responsible for most severe cases and deaths, particularly in Africa, while *P. vivax* is more widespread globally but usually causes a milder form of the disease [1,2,3]. Malaria’s symptoms include fever, chills, headache, nausea, vomiting, and muscle pain. If not treated promptly, it can lead to severe complications such as anaemia, cerebral malaria, and organ failure. Diagnosing malaria involves detecting the presence of the parasite in the blood, typically through microscopic examination or rapid diagnostic tests [2,3]. Malaria is a global health issue, affecting millions of people each year. According to the World Health Organization (WHO), in 2020, there were an estimated 241 million malaria cases and 627,000 malaria deaths worldwide [4]. The disease is endemic in many parts of Africa, Asia, Latin America, and the Middle East. Sub-Saharan Africa bears the heaviest burden, accounting for approximately 95% of malaria cases and 96% of malaria deaths. Children under five and pregnant women are particularly vulnerable to the disease. The human toll of malaria is staggering. It is one of the leading causes of morbidity and mortality in tropical and subtropical regions [5]. Every year, hundreds of thousands of people die from malaria, most of them young children. The loss is not only in terms of lives but also in terms of the potential contributions of those who succumb to the disease. The frequent bouts of illness also lead to significant suffering and disrupt the daily lives and livelihoods of affected individuals and communities. Malaria burdens endemic countries with a heavy economic burden [6]. The costs include healthcare expenditures for diagnosis and treatment, lost productivity due to illness and death, and expenses related to preventive measures such as insecticide-treated nets and indoor residual spraying. The economic impact extends to the national level, with malaria slowing economic growth and development. It is estimated that malaria costs Africa over $12 billion annually in direct costs and much more in lost economic growth [4,7]. Efforts to control and eliminate malaria have significantly progressed over the past few decades. Key strategies include the distribution of insecticide-treated mosquito nets, indoor residual spraying, prompt diagnosis and effective treatment with artemisinin-based combination therapies (ACTs), and preventive therapies for pregnant women and children. Moreover, ongoing research aims to develop new tools, such as more effective vaccines and innovative vector control methods. The RTS, S/AS01 vaccine (Mosquirix^™^) malaria vaccine, recently recommended by the WHO for widespread use among children in Sub-Saharan Africa, represents a significant milestone in the fight against malaria [7]. Despite progress, challenges remain. Additionally, the COVID-19 pandemic has disrupted malaria services, potentially reversing gains made in recent years [8]. Continued investment in research, healthcare infrastructure, and community education is essential to overcome these challenges and move closer to the goal of malaria elimination [3].

PfSEA-1 (Plasmodium falciparum Schizont Egress Antigen-1) is a protein involved in the lifecycle of the malaria-causing parasite Plasmodium falciparum. This protein plays a crucial role in a process known as schizont egress, which is a key step in the replication and spread of the parasite within a human host [9,10]. Understanding PfSEA-1 is important for developing new strategies to combat malaria. Plasmodium falciparum undergoes a complex lifecycle, including stages in the human host and the Anopheles mosquito vector [11]. In humans, the parasite infects red blood cells (RBCs) and replicates within them. The schizont stage is a critical phase in the parasite’s intraerythrocytic cycle, where the parasite multiplies and forms numerous merozoites within a single RBC [12]. The egress of these merozoites from the schizont stage is essential for the propagation of the infection as they invade new RBCs [10]. PfSEA-1 is implicated in the process of schizont egress. It facilitates the rupture of the host RBC membrane and the subsequent release of merozoites into the bloodstream [5]. This release is necessary to continue the parasite’s lifecycle and the progression of malaria symptoms in the host. PfSEA-1 has garnered interest as a potential target for malaria vaccines [13]. Since the protein is critical for the egress of merozoites, blocking its function could effectively halt the parasite’s replication cycle. A vaccine targeting PfSEA-1 could provide immunity by preventing the parasite from escaping infected RBCs, thereby reducing the parasite load and the severity of the disease [13]. In addition to vaccine development, PfSEA-1 is a promising antimalarial drug target. Inhibiting the function of PfSEA-1 could disrupt the lifecycle of *Plasmodium falciparum* at a crucial stage, potentially leading to the development of new therapeutic agents. Given the rising concern over drug resistance in malaria treatment, targeting novel proteins like PfSEA-1 offers a strategic avenue for creating effective treatments. Moreover, PfSEA-1 inhibitors could complement existing therapies, reducing the selective pressure on commonly used antimalarial drugs and slowing resistance emergence. Identifying potent drug candidates against this target not only expands the repertoire of antimalarial strategies but also provides a foundation for future combination therapies, enhancing treatment efficacy and durability [10]. Research into PfSEA-1 is ongoing, with scientists investigating its structure, function, and potential as a therapeutic target. Studies have shown that antibodies against PfSEA-1 can inhibit schizont egress in vitro, suggesting that immune responses targeting this protein could be protective [14]. Additionally, structural studies aim to elucidate the precise mechanisms by which PfSEA-1 facilitates membrane rupture, which could aid in designing inhibitors. Despite its potential, challenges are associated with targeting PfSEA-1 [9]. PfSEA-1′s importance in the replication and spread of the malaria parasite makes it a promising target for vaccine and drug development. Ongoing research aims to unlock the potential of PfSEA-based interventions, which could significantly impact the fight against malaria. However, overcoming the challenges of targeting PfSEA-1 is crucial for developing new and effective treatments [9,13].

**Figure 1:**
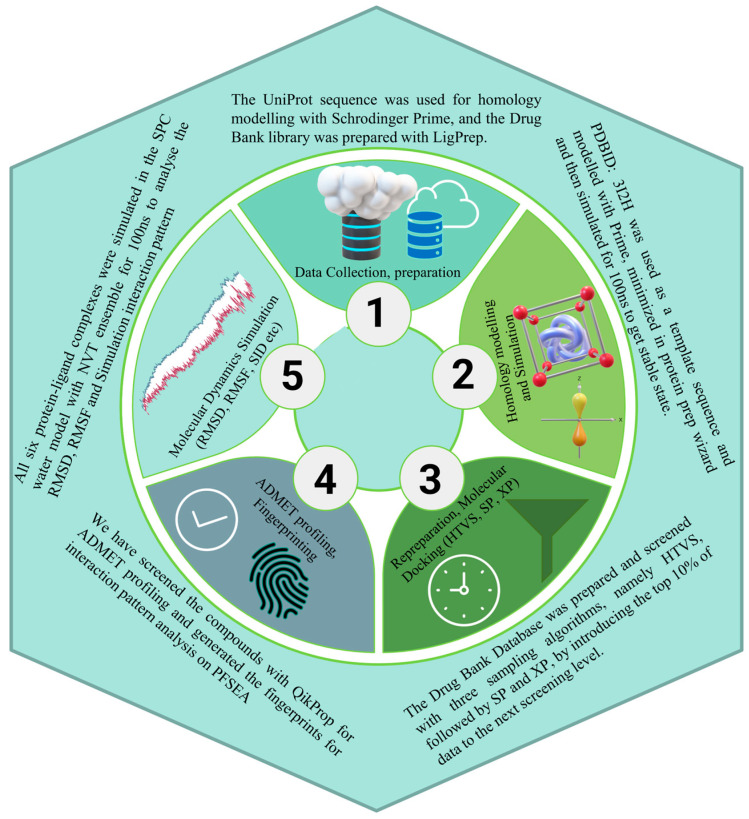
The workflow of the complete study including the data collection, homology modelling, validation, docking, fingerprinting, and MD simulation for reporting the antimalarial compounds.

Malaria remains a global health burden, with Plasmodium falciparum developing increasing resistance to existing antimalarial drugs, including artemisinin-based therapies. The emergence of multidrug-resistant strains highlights the urgent need to identify novel therapeutic targets and drug candidates with unique mechanisms of action. One promising target is Plasmodium falciparum Schizont Egress Antigen-1 (PfSEA-1), a protein critical for the parasite’s lifecycle, making it an attractive focus for drug development. In this study, we aimed to identify potential inhibitors targeting PfSEA-1 using a computational drug discovery pipeline. We first modelled the PfSEA-1 structure and performed 50 ns molecular dynamics (MD) simulations to obtain a stable conformation. Next, we conducted a virtual screening of the entire DrugBank library to identify promising drug candidates based on binding interactions. The top-ranking compounds underwent density functional theory (DFT) analysis for electronic property evaluation and pharmacokinetics screening to assess their drug-likeness. Subsequently, the most promising compounds were subjected to a 100 ns MD simulation in an aqueous environment, followed by binding free energy calculations using the same trajectory to confirm stability and interaction strength. By integrating molecular docking, DFT, MD simulations, and binding free energy analysis, as shown in Figure 1, this study provides a comprehensive computational approach to identifying novel PfSEA-1 inhibitors. Our findings highlight potential drug candidates and contribute to overcoming the persistent challenge of antimalarial drug resistance, paving the way for further experimental validation.

## 2. Results

### 2.1. Protein Structure Preparation and Validation

**Figure 2:**
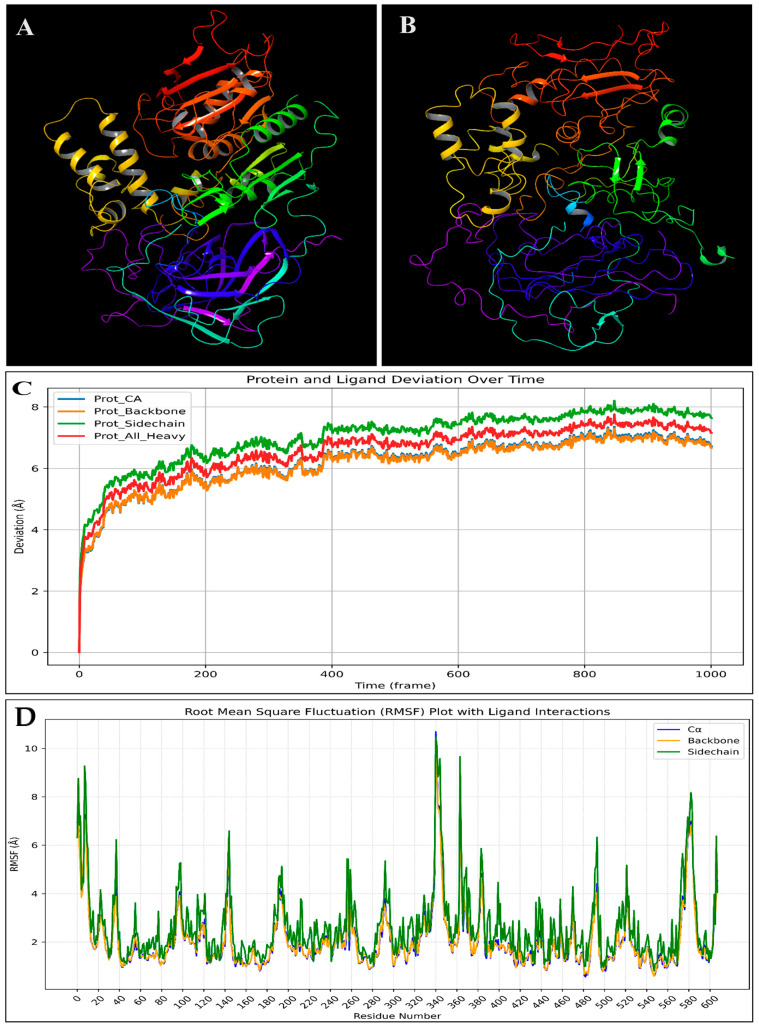
The modelled 3D structures of the protein (**A**) before the molecular dynamics simulation and (**B**) after the 50 ns molecular dynamics simulation. The graphs show the MD simulation results, where (**C**) shows the root-mean-square deviation (RMSD) and (**D**) shows the root-mean-square fluctuation (RMSF) of the PFSEA during 50 ns molecular dynamics simulations.

The energy profile of the PfSEA-1 protein, both before and after a 50-nanosecond molecular dynamics (MD) simulation, reveals significant changes in various energy components. Initially, the system’s total and potential energy was 11,200 kcal/mol. However, after the 50-nanosecond MD simulation, the total and potential energy drastically decreased to −55,700 kcal/mol, indicating a significant stabilisation of the protein structure during the simulation. The bond stretch energy slightly increased from 537 kcal/mol to 563 kcal/mol, suggesting minor adjustments in bond lengths during the simulation. Conversely, the angle bending energy decreased notably from 2960 kcal/mol to 1550 kcal/mol, reflecting a relaxation and optimisation of bond angles within the protein structure. Similarly, the torsion angle energy was substantially reduced from 2120 kcal/mol to 885 kcal/mol, indicating improved torsional conformations post-simulation. The restraining energy for torsions remained at 0 kcal/mol before and after the simulation, implying that no additional restraints were applied to torsional angles (Table 1). The 1,4 Lennard-Jones energy, which accounts for non-bonded interactions within the protein, decreased from 3680 kcal/mol to 2860 kcal/mol, showing enhanced non-bonded interactions. The 1,4 electrostatic energy also slightly reduced from 869 kcal/mol to 846 kcal/mol, suggesting better electrostatic stabilisation. The Lennard-Jones energy, representing van der Waals interactions, significantly decreased from −2170 kcal/mol to −9960 kcal/mol, indicating a more favourable interaction landscape post-simulation. The electrostatic energy, crucial for understanding the overall charge interactions within the protein, dramatically decreased from −2920 kcal/mol to −53,400 kcal/mol, highlighting a significant stabilisation of electrostatic interactions (Table 1). The system’s temperature and total kinetic energy were reported as 0.000 K and 0.00 kcal/mol, respectively, both before and after the simulation, likely due to the data being extracted from an average or snapshot where kinetic energy contributions were not included in this specific analysis. Additionally, the hydrogen bond energy remained at 0.00 kcal/mol throughout, suggesting either a lack of hydrogen bond consideration in this dataset or stable hydrogen bonding interactions that did not change in energy. The MD simulation led to a significant stabilisation of the PfSEA-1 protein, as evidenced by the substantial decreases in total potential, Lennard-Jones, and electrostatic energies. These changes reflect the protein’s adaptation to a more energetically favourable conformation, indicating successful simulation and potential insights into the protein’s dynamic behaviour and stability. During the 50-nanosecond molecular dynamics (MD) simulation of the homology-modelled PfSEA-1 protein, extensive fluctuations exceeding 3 Å were observed in several residues, highlighting regions of pronounced mobility and potential structural flexibility. The N-terminal region, including residues GLU3, MET2, GLU9, LEU10, PHE11, CYS12, TYR13, PRO7, ASN8, ASN4, LYS5, TYR6, ASN25, and GLU24, exhibited significant flexibility, suggesting dynamic interactions or structural adaptations at the protein’s outset. At the C-terminal end, residues such as SER1237, ASN1236, ASP1235, SER1234, ASN1233, THR1232, HIS1231, VAL1230, LEU1228, and ASN1227 showed pronounced mobility, indicating potential involvement in regulatory functions or binding interactions (Figure 2). Within the protein’s interior, residues like GLY189, ASN187, ASN186, ASN188, GLY83, ASP82, and ASP81 displayed internal flexibility, likely influencing the protein’s stability or conformational changes. Residues forming internal secondary structures, including LEU516, VAL519, PHE517, ILE680, HIS682, CYS681, and CYS672, also exhibited dynamic behaviour, suggesting flexibility within structured elements crucial for the protein’s overall stability and function (Figure 2). Moreover, residues clustered in specific regions, such as ASP889, ILE890, HIS891, ASN893, ASN894, ASN908, ASP897, TYR911, and LEU910, displayed significant flexibility, potentially indicating regions critical for the protein’s functional motions or interactions with ligands or substrates. In loops or exposed areas, residues like ARG338, TYR337, THR340, LYS339, and GLY341 showed flexibility, suggesting their involvement in dynamic interactions or conformational changes that affect the protein’s overall structure and function (Figure 2). Understanding these dynamic residues is essential for deciphering the protein’s functional mechanisms, as they provide insights into how the protein may adapt its structure to perform specific biological tasks or interact with other molecules. The observed fluctuations underscore the importance of these residues in maintaining the protein’s flexibility and adapting to its functional requirements.

### 2.2. Molecular Docking Studies

**Figure 3:**
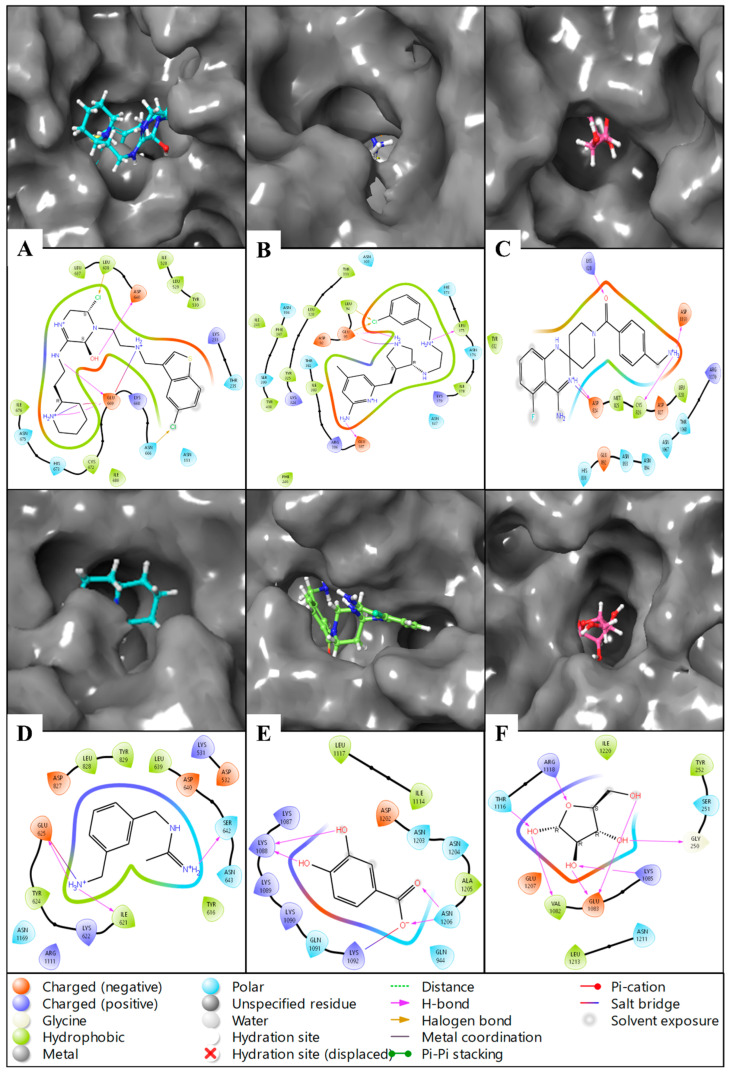
A 3D and 2D ligand interaction diagram of PfSEA-1 with (**A**) Chlobenethyzenol, (**B**) Ametchomine, (**C**) Amiflupipquamine, (**D**) Ambenzyne, (**E**) Dihycid, and (**F**) Alparabinos. The legend shows the residue and interaction types.

We started our study by modelling the protein and preparing it, followed by getting a stable structure by MD simulation, and again preparing it to have the best minimised state, which was further used to generate the grid, followed by conducting molecular docking studies. The molecular docking studies of the Drug Bank compounds with PfSEA-1 have resulted in six unique compounds after being docked and filtered by HTVS, SP and XP and postprocessing by Prime MM\GBSA. At the initial stage, we passed the whole ligand library, and the top 10% of HTVS ligands were passed to redock with SP, and the top 10% of SP were passed to redock with XP. All 100% XP results computed with four poses were passed to pose-process with Prime MM\GBSA, which resulted in nine drug candidates (three duplicates of six unique candidates), which further processed and identified the six candidates with good scoring and validated with the various studies. The detailed interaction studies between the protein PfSEA and six ligands—Alparabinos, Dihycid, Ambenzyne, Amiflupipquamine, Ametchomine, and Chlobenethyzenol—have provided comprehensive insights into their binding affinities and interaction specifics, indicated by their docking scores and other binding parameters. The PfSEA-Chlobenethyzenol complex demonstrated a strong binding affinity with a docking score of −8.107 kcal/mol. This interaction featured three hydrogen bonds involving the GLU669 residue with N+H2 and NH atoms and the ASP640 residue with the OH atom. Additionally, two salt bridges were formed with GLU669 residue, two N+H2 atoms, and two halogen bonds involving LEU638 and ASN666 residues with two different Cl atoms of Chlobenethyzenol. The MMGBSA dG Bind Coulomb energy was −445.18 kcal/mol, reflecting significant electrostatic interactions, and the MMGBSA dG Bind Hbond was −1.95 kcal/mol. The ligand efficiency and ligand efficiency sa were −0.262 and −0.822, respectively, indicating favourable binding characteristics (Figure 3A, Table 2). For the PfSEA–Ametchomine complex, the docking score was −7.005 kcal/mol. The binding involved four hydrogen bonds among the LYS1088 residue with two OH atoms and the ASN1206 residue with two O atoms. A salt bridge was formed between the LYS1092 residue and the O atom of the ligand. The MMGBSA dG Bind Coulomb energy was −319.81 kcal/mol, and the MMGBSA dG Bind Hbond was −3.53 kcal/mol. The ligand efficiency and ligand efficiency sa were −0.269 and −0.798, respectively (Figure 3B, Table 2). The PfSEA–Amiflupipquamine complex showed a docking score of −6.94 kcal/mol. The interactions included hydrogen bonds with ILE621 and GLU625 residues with the N+H3 atom and the SER642 residue with the N+H2 atom. A salt bridge was also formed with the GLU625 residue and the N+H3 atom of the ligand. The MMGBSA dG Bind Coulomb energy was −385.43 kcal/mol, and the MMGBSA dG Bind Hbond was −2.69 kcal/mol. The ligand efficiency and ligand efficiency sa were −0.257 and −0.771, respectively (Figure 3C, Table 2). In the case of the PfSEA–Ambenzyne complex, the docking score was −6.505 kcal/mol. This interaction involved hydrogen bonds between the CYS826 and ASP1190 residues with the N+H3 atom, the ASP824 residue with a N+H atom, and the LYS628 residue with an O atom. A salt bridge was also observed between the ASP824 residue and the N+H atom of the ligand. The MMGBSA dG Bind Coulomb energy was −375.08 kcal/mol, and the MMGBSA dG Bind Hbond was −3.04 kcal/mol. The ligand efficiency and ligand efficiency sa were −0.5 and −1.177, respectively (Figure 3D, Table 2). The PfSEA–Dihycid complex had a docking score of −6.225 kcal/mol. The binding involved three hydrogen bonds connecting the GLU93, LEU175, and GLU387 residues with three different N+H2 atoms and a salt bridge with the GLU93 residue and a N+H2 atom. Two halogen bonds were identified with the GLU93 and LEU94 residues and the Cl atom of the ligand. Interestingly, the MMGBSA dG Bind Coulomb energy was 78.17 kcal/mol, suggesting an unfavourable electrostatic contribution, while the MMGBSA dG Bind Hbond was −3.87 kcal/mol. The ligand efficiency and ligand efficiency sa were −0.566 and −1.258, respectively (Figure 3E, Table 2). Finally, the PfSEA–Alparabinos complex showed a docking score of −4.481 kcal/mol. The interaction involved three hydrogen bonds between the GLU669 residue and N+H2 and NH atoms and between the ASP640 residue and the OH atom, as well as two salt bridges with GLU669 residue and two N+H2 atoms. Two halogen bonds involving LEU638 and ASN666 residues were also noted with two different Cl atoms of Alparabinos. The MMGBSA dG Bind Coulomb energy was −40.44 kcal/mol, and the MMGBSA dG Bind Hbond was −4.65 kcal/mol. The ligand efficiency and ligand efficiency sa were −0.448 and −0.965, respectively (Figure 3F, Table 2).

### 2.3. Molecular Interaction Fingerprints

**Figure 4:**
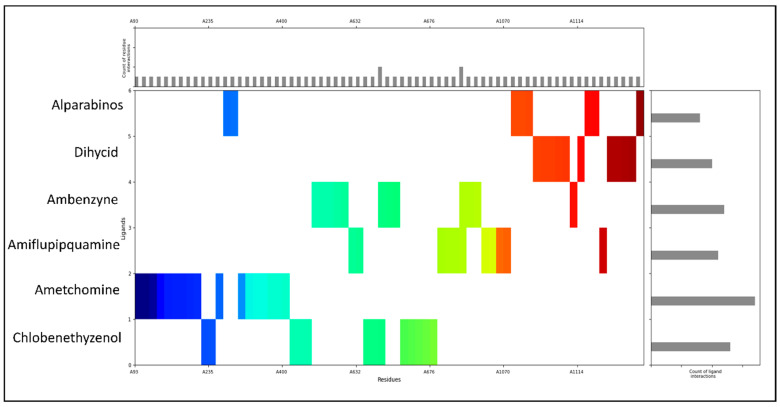
Molecular interaction fingerprints. The main plot is coloured to show the N to C terminal of the protein, the left plot shows the count of ligand interactions, and the upper plot shows the count of interacting residues.

The molecular interaction fingerprints (MIFs) computed for the docked protein–ligand complexes of PfSEA-1 with the six ligands reveal key interactions crucial for stabilising these complexes. Here is the count of residues found during fingerprinting: 11ASN, 10LYS, 8ASP, 8LEU, 6ILE, 6TYR, 5GLU, 4ARG, 2CYS, 2PHE, 2THR, 1ALA, 1GLY, 1HIE, 1MET, and 1SER. Analysis of the residue counts highlights diverse interactions involving polar, hydrophobic, and charged residues, underscoring the multifaceted nature of protein–ligand binding. ASN (asparagine) emerges prominently among the identified residues, with 11 occurrences across the complexes. Asparagine residues typically contribute polar interactions through their side-chain amide groups, forming hydrogen bonds with ligand atoms such as oxygen or nitrogen. This interaction stabilises the complex by facilitating specific, directional bonds contributing to binding affinity and specificity (Figure 4). LYS (lysine) residues, totalling 10, are notable for their positively charged side chains, which often engage with salt bridges with negatively charged ligand atoms or stabilise the complex through hydrogen bonding. These interactions are pivotal in orienting the ligand within the binding pocket and can significantly influence the binding affinity by electrostatic attraction. ASP (aspartic acid) and GLU (glutamic acid), with eight and five occurrences, respectively, contribute negatively charged side chains that can form salt bridges with positively charged ligand atoms. These interactions contribute to the electrostatic complementarity between the protein and ligand, enhancing the stability of the complex (Figure 4). Hydrophobic interactions are also evident, with residues like LEU (leucine), ILE (isoleucine), TYR (tyrosine), PHE (phenylalanine), and others participating. These residues, totalling eight, six, six, two, and two occurrences, respectively, contribute to the hydrophobic core of the binding site. They interact with non-polar regions of the ligand, shielding them from the aqueous environment and contributing to the overall stability of the complex through van der Waals forces. ARG (arginine), CYS (cysteine), THR (threonine), and other residues, although less frequent, also play crucial roles. Depending on the ligand’s charge distribution, ARG residues (four occurrences) can form salt bridges or hydrogen bonds. CYS (two occurrences) might form disulfide bridges or stabilise through polar interactions. THR (two occurrences) contributes to its polar side chain, facilitating hydrogen bonding or van der Waals interactions.

### 2.4. Pharmacokinetic Studies

The QikProp-based pharmacokinetic properties of the six drugs—Alparabinos, Dihycid, Ambenzyne, Amiflupipquamine, Ametchomine, and Chlobenethyzenol—provide detailed insights into their potential as therapeutic agents [15,16,17]. Chlobenethyzenol has notable pharmacokinetic properties with a surface area (SASA) of 312.149, a relatively moderate molecular weight (mol MW) of 150.131, and a low dipole moment of 1.757, suggesting a balanced distribution of charge. It has a fraction of hydrophobic surface area (FOSA) of 127.898 and a fraction of ionisable surface area (FISA) of 184.251, indicating significant hydrophobic and ionisable regions. The polar surface area (PSA) is 97.786, within the standard range, suggesting good absorption and permeability. Chlobenethyzenol permeability across the blood–brain barrier (QPlogBB) is −1.084, implying limited central nervous system (CNS) penetration. It also has a high passive permeability (QPPCaco) of 177.276, indicating good intestinal absorption. The predicted human oral absorption is 56.972%, with a moderate lipophilicity (QPlogPo/w) of −1.745, indicating a balanced hydrophilic–lipophilic profile (Table 3). Ametchomine shows a mol MW of 154.122, fitting well within the acceptable range. It has an SASA of 331.243 and a higher dipole moment of 5.953, suggesting a more significant charge distribution. The FISA is 206.617, indicating a high fraction of ionisable surface area, and the PSA is 93.526. The QPlogBB is −1.223, suggesting poor CNS penetration, while the QPPCaco is 27.552, indicating moderate intestinal absorption. The human oral absorption is 52.903%, with a QPlogPo/w of 0.031, indicating balanced hydrophilic and lipophilic properties (Table 3). Amiflupipquamine has a lower mol MW of 177.249, an SASA of 448.687, and a dipole moment of 4.115. It has a moderate FISA of 145.123 and a PSA of 73.945, suggesting good absorption properties. The QPlogBB of −0.653 suggests moderate CNS penetration, and the QPPCaco of 103.895 indicates good intestinal absorption. The predicted human oral absorption is 64.615%, with a QPlogPo/w of 0.269, indicating slightly hydrophilic properties (Table 3). Ambenzyne features a mol MW of 367.425, an SASA of 641.741, and a higher dipole moment of 5.54. It has an FISA of 163.364 and a PSA of 99.369, indicating good absorption and permeability. The QPlogBB of −0.807 suggests limited CNS penetration, while the QPPCaco of 69.763 indicates good intestinal absorption. The human oral absorption is 71.754%, with a QPlogPo/w of 2.017, suggesting balanced hydrophilic–lipophilic properties (Table 3). Dihycid has a mol MW of 373.928, an SASA of 598.359, and a dipole moment of 3.578. It has an FISA of 114.794 and a PSA of 78.123, suggesting good absorption and permeability. The QPlogBB of −0.032 indicates poor CNS penetration, while the QPPCaco of 12.532 suggests poor intestinal absorption. Human oral absorption is 54.65%, with a QPlogPo/w of 1.375, indicating moderate lipophilicity (Table 3). Alparabinos has a higher mol MW of 484.486, a large SASA of 833.794, and a dipole moment of 3.471. It has an FISA of 88.112 and a PSA of 74.425, indicating good absorption properties. The QPlogBB of 0.144 suggests potential CNS penetration, while the QPPCaco of 22.441 indicates poor intestinal absorption. The human oral absorption is 70.402%, with a QPlogPo/w of 3.292, indicating significant lipophilicity. The pharmacokinetic profiles of these drugs suggest that while most have good absorption and permeability, factors such as CNS penetration, intestinal absorption, and lipophilicity vary significantly. These insights are critical for understanding the potential efficacy and optimisation needed for each drug candidate.

### 2.5. DFT-Based Compound Optimisation Studies

**Figure 5:**
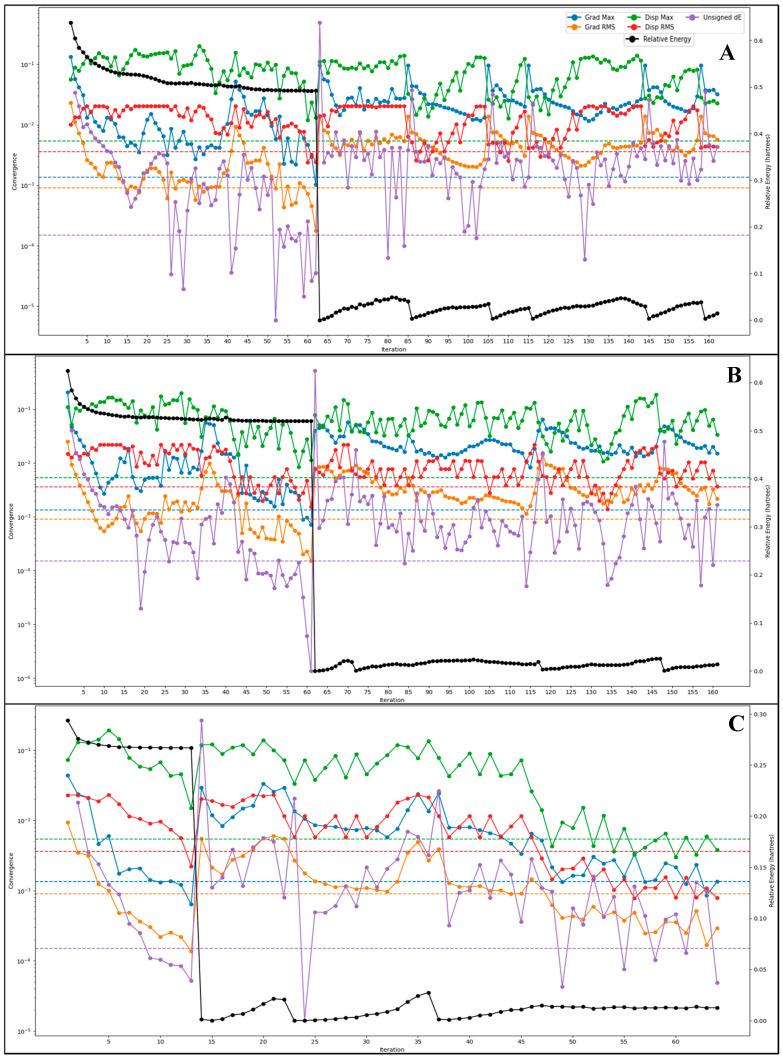

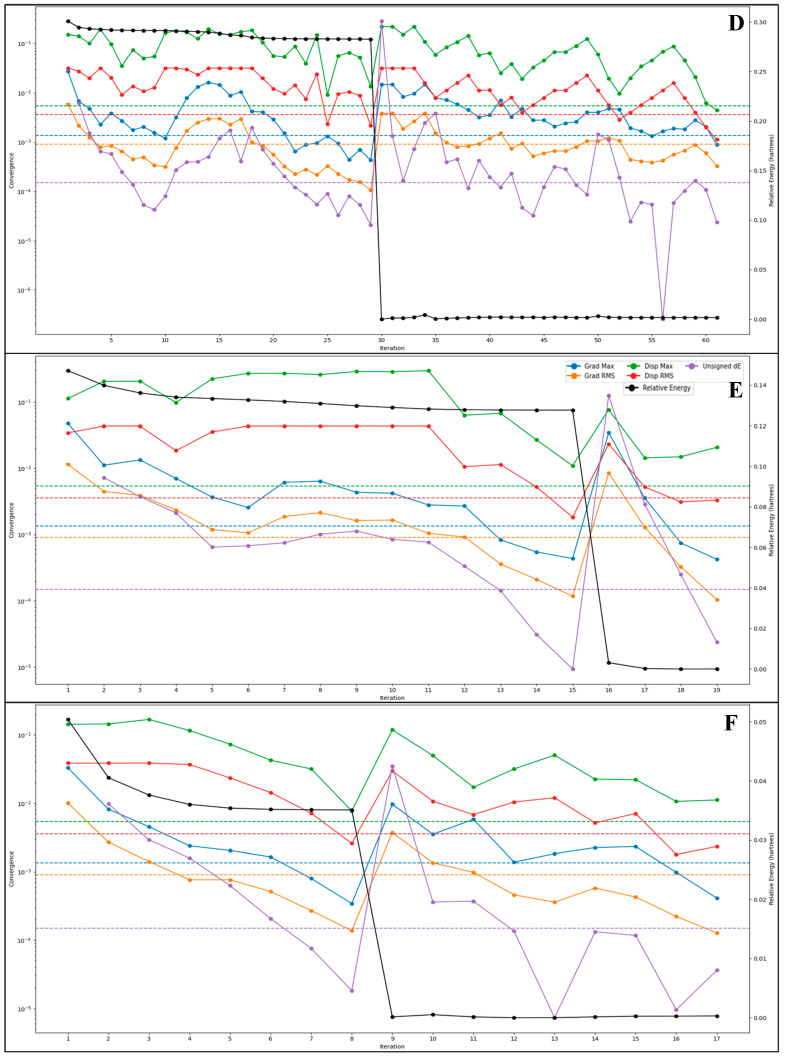
The Jaguar Optimisation tool shows the optimisation results of (**A**) Alparabinos, (**B**) Dihycid, (**C**) Ambenzyne, (**D**) Amiflupipquamine, (**E**) Ametchomine, and (**F**) Chlobenethyzenol where black shows the relative energy converging in each case to show that the ligands are stable.

The quantum mechanical optimisation of the six drug candidates—Alparabinos, Dihycid, Ambenzyne, Amiflupipquamine, Ametchomine, and Chlobenethyzenol—utilised the DFT(b3lyp-d3)/SOLV method with a 6-31G** basis set and a spin multiplicity of 1, confirming that all molecules are in their ground singlet states. This computational approach allowed for a comprehensive examination of their electronic and vibrational characteristics, providing critical insights into their potential efficacy and stability as therapeutic agents. The energy calculations reveal significant differences between gas and solution phase environments, emphasising the solvation effects on molecular stability. Alparabinos, with a gas phase energy of −2525.621655 au and a solution phase energy of -2526.099577 au, demonstrates the highest solvation energy at −299.9 kcal/mol (Figure 5). This substantial stabilisation suggests that Alparabinos benefits greatly from solvation, enhancing its potential bioavailability and stability in aqueous environments. In stark contrast, Chlobenethyzenol shows minimal solvation energy at −21.84 kcal/mol, indicating that it experiences less stabilisation in solution, which could affect its solubility and overall effectiveness. The HOMO (Highest Occupied Molecular Orbital) and LUMO (Lowest Unoccupied Molecular Orbital) energies provide further insights into the electronic properties and reactivity of these drug candidates (Figure 5). Alparabinos exhibits an HOMO energy of −0.23864 au and an LUMO energy of −0.05185 au, while Chlobenethyzenol has an HOMO energy of −0.26614 au and an LUMO energy of 0.06226 au. The smaller HOMO–LUMO gap in Chlobenethyzenol implies higher reactivity, making it a potentially more effective compound, albeit possibly less stable. This electronic property could be crucial in interacting with biological targets, potentially enhancing its therapeutic activity. Vibrational frequency analysis shows these molecules’ dynamic behaviour and stability (Figure 5). Alparabinos shows the lowest frequency at −107.947742 cm^−1^ and the highest at 3732.961503 cm^−1^, indicating a wide range of vibrational modes and suggesting that it can adopt multiple conformations. Ambenzyne, with a notably low frequency of −247.25293 cm^−1^, suggests possible instability or significant conformational flexibility, which could impact its interaction with biological targets and overall stability. The presence of negative frequencies, particularly in Alparabinos (seven negative frequencies), contrasts with Ametchomine and Chlobenethyzenol, which have no negative frequencies, indicating higher stability for the latter two compounds. Thermodynamic properties such as zero-point energy, entropy, enthalpy, and free energy further elucidate these drug candidates’ stability and potential efficacy. Alparabinos, with a zero-point energy of 353.885 kcal/mol, has the highest intrinsic energy among the candidates, reflecting its inherent stability. Free energy calculations at 298.15 K and 1 atm show that Alparabinos has a free energy of -31.838737 kcal/mol, suggesting that it is thermodynamically stable and potentially efficacious in physiological conditions. Ametchomine, with a free energy of −20.953374 kcal/mol, also indicates good stability, albeit lower than Alparabinos (Figure 5). Electrostatic potential (ESP) analysis provides a detailed view of the charge distribution within these molecules. Alparabinos has an ESP max of 250.43 kcal/mol and an ESP min of 96.36 kcal/mol, indicating significant electrostatic interactions that could enhance binding to biological targets. Ametchomine exhibits distinct positive and negative charge regions with an ESP min of −176.46 kcal/mol and an ESP max of 12.88 kcal/mol, which may influence its binding affinity and specificity. The average local ionisation energy (ALIE) descriptors offer insights into the ease of electron removal from these molecules. Alparabinos shows an ALIE mean of 280.9 kcal/mol, indicating moderate stability and reactivity, while Chlobenethyzenol, with an ALIE mean of 276.97 kcal/mol, demonstrates a higher degree of uniformity in electronic properties, reflected by its lower variance (Figure 5). This consistency in electronic behaviour suggests that Chlobenethyzenol may have predictable interactions with biological targets, enhancing its potential as a drug candidate. The quantum mechanical optimisation results provide an in-depth understanding of the electronic, vibrational, and thermodynamic properties of Alparabinos, Dihycid, Ambenzyne, Amiflupipquamine, Ametchomine, and Chlobenethyzenol. These properties determine their stability, reactivity, and potential as therapeutic agents. Alparabinos and Chlobenethyzenol, in particular, exhibit unique characteristics that could influence their bioavailability and effectiveness, making them promising candidates for further drug development. The comprehensive analysis underscores the importance of such detailed computational studies in the rational design and optimisation of new pharmaceuticals.

### 2.6. Molecular Dynamics Simulation

The System Builder generated 75070 atoms for PfSEA-1 in complex with Chlobenethyzenol, 75077 atoms for PfSEA-1 in complex with Ametchomine, 75054 atoms for PfSEA-1 in complex with Amiflupipquamine, 75030 atoms for PfSEA-1 in complex with Ambenzyne, 74968 atoms for PfSEA-1 in complex with Dihycid, and 75028 atoms for PfSEA-1 in complex with Alparabinos. The built file was loaded and used for a 100 ns production run, and the trajectories were analysed to compute the deviation, fluctuation, and simulation interactions. Below are the detailed results.

#### 2.6.1. Root-Mean-Square Deviation (RMSD)

**Figure 6:**
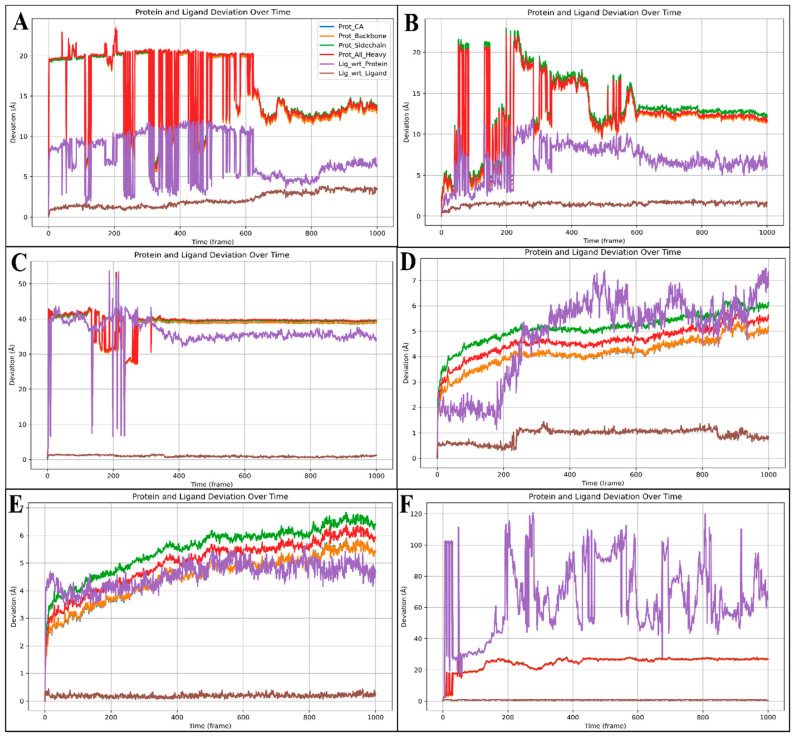
The root-mean-square deviation (RMSD) of PfSEA-1 and (**A**) Chlobenethyzenol, (**B**) Ametchomine, (**C**) Amiflupipquamine, (**D**) Ambenzyne, (**E**) Dihycid, and (**F**) Alparabinos during 100 ns molecular dynamics simulation in the TIP3P water model.

Root-mean-square deviation (RMSD) measures the average distance between atoms of superimposed proteins, indicating structural stability and conformational changes during molecular dynamics (MD) simulations. Low RMSD values suggest a stable structure, whereas high values indicate significant deviations, reflecting flexibility or conformational shifts. RMSD is crucial for assessing the reliability and stability of protein–ligand complexes in drug design studies. The complex involving the protein PfSEA-1 and ligand Alparabinos initially showed significant deviations, with the protein at 19.33 Å and the ligand at 7.40 Å at 0.10 ns. By 62.30 ns, the deviations were 19.92 Å for the protein and 10.96 Å for the ligand. Stability was observed after 63.30 ns, with 14.97 Å (protein) and 5.87 Å (ligand) deviations. At 100 ns, the protein deviation was 12.92 Å and the ligand was 6.39 Å, indicating initial instability followed by stabilisation (Figure 6A). For the complex with the protein PfSEA and the ligand Dihycid, there was a significant deviation in the early stages: 2.61 Å (protein) and 2.44 Å (ligand) at 3.90 ns, increasing to 13.55 Å (protein) and 7.16 Å (ligand) by 59.20 ns. Stability was achieved after 59.80 ns, with the protein at 11.92 Å and the ligand at 7.48 Å, remaining relatively constant at 11.60 Å (protein) and 7.17 Å (ligand) at 100 ns (Figure 6B). In the PfSEA and Ambenzyne complex, initial deviations were 2.67 Å (protein) and 2.12 Å (ligand) at 0.10 ns, increasing to 39.20 Å (protein) and 39.56 Å (ligand) by 31.30 ns. The complex stabilised from 34.00 ns, with the protein at 139.65 Å and the ligand at 37.17 Å, maintaining stability at 100 ns with deviations of 38.88 Å (protein) and 33.73 Å (ligand) (Figure 6C). The PfSEA and Amiflupipquamine complex showed initial deviations of 3.16 Å (protein) and 1.87 Å (ligand) at 7.60 ns, with consistent performance throughout the simulation. At 100 ns, the protein deviated by 5.13 Å and the ligand deviated by 7.06 Å, indicating acceptable stability after the initial fluctuations (Figure 6D). For the PfSEA and Ametchomine complex, the protein deviation started at 3.21 Å and the ligand started at 4.04 Å at 8.20 ns. At 100 ns, the deviations were 5.38 Å (protein) and 4.47 Å (ligand), showing stability after initial fluctuations (Figure 6E). Lastly, the PfSEA and Chlobenethyzenol complex exhibited initial deviations of 17.79 Å (protein) and 27.92 Å (ligand) at 3.20 ns. By 100 ns, the deviations were 26.59 Å (protein) and 70.24 Å (ligand), indicating significant deviations and less stability compared to other complexes (Figure 6F). From the analysis, the Amiflupipquamine and Ametchomine complexes demonstrated the best stability and lowest deviations, suggesting that they are the most promising candidates for further drug development. The Alparabinos complex also showed potential after the initial instability was resolved. Conversely, the Ambenzyne and Chlobenethyzenol complexes exhibited significant instability, indicating they are less suitable without substantial modifications.

#### 2.6.2. Root-Mean-Square Fluctuations (RMSF)

**Figure 7:**
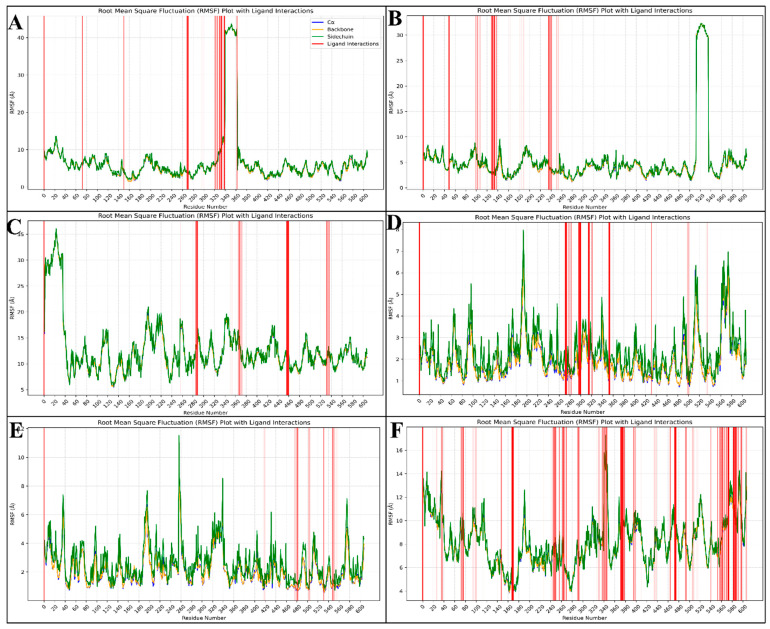
The root-mean-square fluctuations (RMSFs) of the PfSEA-1 and (**A**) Chlobenethyzenol, (**B**) Ametchomine, (**C**) Amiflupipquamine, (**D**) Ambenzyne, (**E**) Dihycid, and (**F**) Alparabinos during 100 ns molecular dynamics simulation in the TIP3P water model.

Root-mean-square fluctuation (RMSF) measures the average deviation of each atom or residue from its mean position throughout an MD simulation. RMSF provides insights into the flexibility and dynamic behaviour of different molecule parts. High RMSF values indicate regions with greater flexibility, while low values suggest more rigid, stable regions. This metric is essential for understanding the dynamic properties of protein–ligand complexes and identifying potential binding sites. The complex of PfSEA-1 and Chlobenethyzenol has shown some residues fluctuating, and the top fluctuated residues are ASP827, CYS826, LEU828, MET825, ASP824, GLU822, ASN823, TYR829, LEU821, ASN830, LEU831, LEU820, GLY815, HIS837, GLU819, ILE816, LYS832, TYR817, ASP818, MET833, ASP835, LEU836, and HIS834, and at the same time, many residues have interacted to make the complex stable, these being HIS837, CYS681, HIS682, MET677, ILE676, ASN675, HIS673, CYS672, GLU669, LYS668, ASP117, ASN666, ASP665, LEU638, LEU637, LEU641, ASN664, ASP640, LEU639, SER642, ILE528, ASN643, LYS233, GLU644, LEU529, TYR530, and LYS531 (Figure 7A). The complex of PfSEA-1 and Ametchomine has shown some residues fluctuating, and the top fluctuated residues are GLU1159, PRO1157, ILE1158, TYR1160, GLY1156, LYS1161, LYS1162, ILE1163, ILE1155, TYR1164, ILE1154, LEU1165, HIS1153, LYS1166, PRO1152, SER1151, ASN1167, ASP1149, and LEU1168, and at the same time, many residues have interacted to make the complex stable, these being ALA144, GLU335, ASN145, ASN147, GLN332, TYR331, ASP381, LEU94, ASN130, ASP148, THR382, GLU93, GLU152, LEU328, SER385, ILE383, ARG386, LYS182, ASN396, LYS179, SER399, ASN176, HIS173, TYR400, LEU175, GLU387, PHE397, ILE178, and PHE246 (Figure 7B). The complex of PfSEA-1 and Amiflupipquamine has shown some residues fluctuating, and the top fluctuated residues are ASN25, GLY26, GLU24, GLU27, ILE23, ILE22, GLU28, GLU21, ASN20, LYS29, GLU36, ASP37, ILE19, TYR30, ASP35, VAL31, and ASN18, and at the same time, many residues have interacted to make the complex stable, these being GLU819, LEU516, GLU822, ASN823, TYR632, ASP824, HIS891, CYS826, ASN830, ASN893, MET825, VAL631, GLU892, ASP827, ASN894, ILE629, LEU828, TYR829, ASN630, LYS628, ILE895, ARG1066, THR1068, ASP1190, SER1170, ASN898, LEU1168, ASN1069, ASN1067, ARG1070, ASN1167 (Figure 7C). The complex of PfSEA-1 and Ambenzyne has shown some residues fluctuating, and the top fluctuated residues are THR340, LYS339, LEU1228, ASP1149, ILE1220, GLY341, ASN1227, HIS1229, VAL1221, LYS1218, and ARG338, and at the same time, many residues have interacted to make the complex stable, these being VAL658, ASN659, ASN664, ASN657, ASN1171, TYR829, ASN643, SER642, LEU641, LYS531, ILE621, LEU828, TYR616, LEU639, ASP640, ASP532, ASP998, ASN1169, TYR1108, GLU625, PHE617, ASP827, ARG1111, and LYS1110 (Figure 7D). The complex of PfSEA-1 and Dihycid has shown some residues fluctuating, and the top fluctuated residues are LEU516, PHE517, ASP81, GLY341, GLU518, HIS682, VAL519, and GLU342, and at the same time, many residues have interacted to make the complex stable, these being ILE1114, ALA1205, MET945, ASP1202, LEU1086, ASN1203, ASN1209, ASN1204, GLU1201, HIS1210, GLN944, ASN1206, LYS1090, SER942, ASN1112, LYS1088, GLN1091, LYS1092, and ASN1167 (Figure 7E). The complex of PfSEA-1 and Alparabinos has shown some residues fluctuating, and the top fluctuated residues are ASP818, GLY815, GLU819, ILE816, and TYR817, and at the same time, many residues have interacted to make the complex stable, these being ASP818, GLY815, GLU819, ILE816, TYR817, ASP37, LEU821, LEU820, and many more residues (Figure 7F).

The fluctuating residues identified in the PfSEA-1 and Chlobenethyzenol complex include ASP827, CYS826, LEU828, and others. These residues are primarily located in flexible regions of the protein, which are often loops or termini. Such fluctuations can lead to conformational changes that might affect the binding pocket’s shape and accessibility, potentially impacting the binding affinity and stability of the complex. Conversely, residues such as HIS837, CYS681, and HIS682 stabilised interactions. These residues often form hydrogen bonds, hydrophobic interactions, or salt bridges, which are crucial for maintaining the structural integrity and stability of the complex. The residues showing significant fluctuations in the PfSEA-1 and Ametchomine complex include GLU1159, PRO1157, and ILE1158. Being in more flexible regions, these residues suggest potential conformational adaptability, which could influence ligand binding and the overall stability of the protein–ligand complex. On the stability front, residues like ALA144, GLU335, and ASN145 play key roles in stabilising the complex. These interactions likely contribute to a stable binding interface, reducing the overall flexibility and enhancing the complex’s rigidity. In the PfSEA-1 and Amiflupipquamine complex, fluctuating residues such as ASN25, GLY26, and GLU24 indicate regions of higher flexibility. These fluctuations could impact the stability by causing transient structural changes, affecting the ligand’s binding efficacy. Residues like GLU819, LEU516, and GLU822 primarily contributed to stabilising interactions. These residues likely engage in essential interactions such as hydrogen bonds and hydrophobic contacts, which help maintain the structural coherence of the complex. The residues THR340, LYS339, and LEU1228 in the PfSEA-1 and Ambenzyne complex showed notable fluctuations. These flexible regions may undergo conformational changes that influence the binding dynamics and stability of the complex. Stabilising residues such as VAL658, ASN659, and ASN664 are critical in maintaining structural stability. These interactions help anchor the ligand firmly within the binding site, thereby reducing the overall flexibility of the complex. The fluctuating residues identified in the PfSEA-1 and Dihycid complex, such as LEU516, PHE517, and ASP81, suggest regions of high flexibility. This flexibility can lead to structural alterations that impact the stability and binding affinity of the complex. Residues like ILE1114, ALA1205, and MET945 contribute significantly to the stability. These residues likely form stable interactions that prevent large conformational changes, ensuring the complex remains stable. In the PfSEA-1 and Alparabinos complex, residues such as ASP818, GLY815, and GLU819 fluctuated highly. The flexibility of these residues could lead to significant conformational changes, potentially impacting the binding stability and affinity. Conversely, residues like ASP818, GLY815, and GLU819 also stabilised the complex, highlighting the dual nature of some residues in both flexibility and stability. Their interactions likely help maintain a balance between flexibility and stability, which is crucial for the proper functioning of the complex. The fluctuation of residues typically indicates protein regions that are more flexible and may undergo conformational changes. These changes can influence the stability of the complex by altering the binding site, thereby affecting the binding affinity and dynamics of the ligand.

#### 2.6.3. Simulation Interaction Diagram (SID) Studies

**Figure 8:**
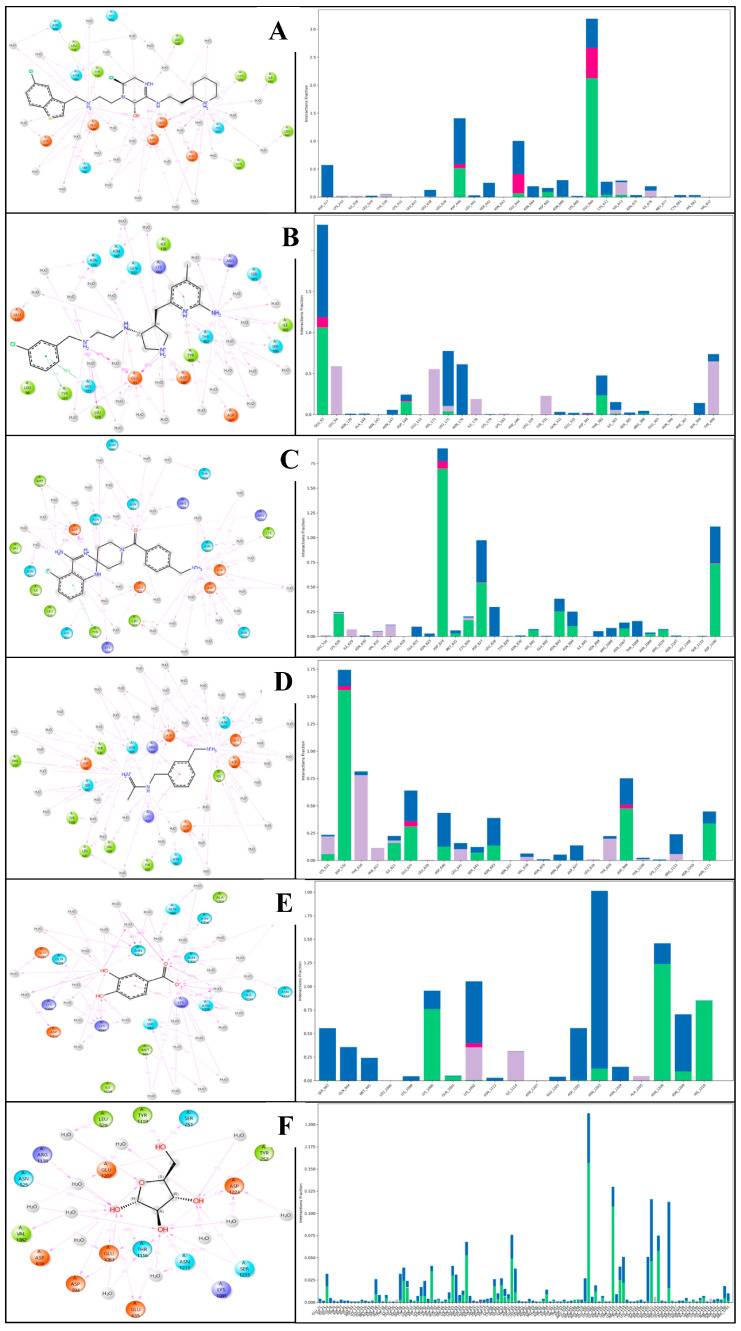
A simulation interaction diagram (SID) and histogram representation of the count of interactions during the simulation among PfSEA-1 and (**A**) Chlobenethyzenol, (**B**) Ametchomine, (**C**) Amiflupipquamine, (**D**) Ambenzyne, (**E**) Dihycid, and (**F**) Alparabinos during 100 ns molecular dynamics simulation in the TIP3P water model.

A simulation interaction diagram visually represents the interactions between proteins and ligands during an MD simulation. It typically includes various plots such as hydrogen bonds, hydrophobic interactions, water bridges, and ionic interactions over time. This diagram helps identify key interaction patterns and stability, providing insights into binding mechanisms and aiding in optimising drug candidates by highlighting critical interaction sites and their dynamics throughout the simulation. The complex with the protein PfSEA-1 and the ligand Alparabinos shows interactions between hydrogen bonds: GLU644, ASP665, GLU669, HIS673, CYS672, ASP640, ASN664, ASN666, SER642, ASP117, CYS681, and ILE676 residues interact with water molecules alongside two N+H2 atoms; the ASP640 residue interacts via the N+H atom; ASP640 and ASN666 residues, along with water molecules and GLU 669 residue, interact via the NH atom; and the OH atom, alongside ASP640, GLU669, LEU638, and SER642 residues, interacts with water molecules along the OH atom. Moreover, a pi-cation contacts the HIS673 residue via the N+H2 atom, and four salt bridges form, interacting with ASP640, GLU644, and HIS673 residues via two N+H2 atoms, and the GLU669 residue interacts with the N+H atom of the ligand Alparabinos (Figure 8A). The protein PfSEA and Dihycid produces many interactions alongside hydrogen bonds amongst GLU93, LEU175, THR382, and ASP148 residues, with three N+H2 atoms and the NH atoms by GLU93 and THR382 residues. Additionally, three pi–pi stacking contacts HIS173, TYR331, and TYR400 residues with two benzene rings; a pi-cation contacts the TYR400 residue by the benzene ring and forms two salt bridges with GLU93 and ASP381 residues with the N+H2 atom of the ligand Dihycid (Figure 8B). Interactions in the complex with protein PfSEA and Ambenzyne formed hydrogen bonds with CYS826, ASP827, ASP1190, ASN1067, LEU828 residues with water molecules via the N+H3 atom; the ASP824 residue interacts via the N+H2 atom; two NH atoms contact LYS628, ASP824, HIS891, and ASN894 residues; the O atom interacts via ASN893, ASN894, ARG1070, GLU822, ASN898, ASN894, THR1068, and ARG1066 residues with water molecules. Further, a pi–pi stacking contacts the TYR632 residue with the benzene ring and forms a salt bridge that contacts the ASP827 residue along the N+H3 atom of the ligand Ambenzyne (Figure 8C). The complex with PfSEA and the ligand Amiflupipquamine interacts with many hydrogen bonds among ASP532, ILE621, GLU625, ASP640, ASN1171,and ASP827 residues with water molecules via the N+H3 atom; SER642, ASP998, ASP532, and ASN643 residues interact with water molecules by the N+H2 atom; and the NH atom interacts with ASN532, LYS531, ASN643, ASN664, and ARG1111 residues with water molecules. Also, three pi-cations contact LYS531 and ARG1111 residues via the benzene ring and the TYR616 residue via the N+H2 atom; and three salt bridges contact ASP532 and GLU625 residue with the N+H3 atom, and the ASP998 residue by the N+H2 atom of the ligand Amiflupipquamine (Figure 8D). The interaction between the protein PfSEA and the ligand Ametchomine shows hydrogen bonds with ASP1202 and ASN1203 residues with water molecules and LYS1090 residues with two OH atoms, as well as ASN1203, ASN1209, HIS1210, ASN1206, ASN1203, MET945, SER942, GLN944, and LYS1092 residues with water molecules via two O atoms; and a pi-cation contacts the LYS1092 residues along the benzene ring of the ligand Ametchomine (Figure 8E). The complex with the protein PfSEA and the ligand Chlobenethyzenol formed hydrogen bonds with four OH atoms along SER251, TYR252, ASP1223, SER1215, LYS1085, ASN1211, GLU635, THR1116, GLU1083, ASP394, TYR1119, ARG1118, and ASP818 residues; and the O atom contacted LEU529 and GLU1083 residues with water molecules (Figure 8F).

The interactions observed in the complexes of the protein PfSEA with various ligands—Alparabinos, Dihycid, Ambenzyne, Amiflupipquamine, and Ametchomine—highlight the diverse binding modes and molecular interactions that play crucial roles in protein–ligand recognition and stabilisation. Hydrogen bonds are pivotal in stabilising protein–ligand complexes by forming specific interactions between the protein’s amino acid residues and the ligands’ functional groups. In the case of PfSEA with Alparabinos, significant hydrogen bonds were observed involving GLU644, ASP665, GLU669, HIS673, CYS672, and ASP640 residues. These interactions facilitate the formation of a stable complex, where GLU669 and ASP640 residues also interact with water molecules, enhancing the binding affinity through additional polar interactions. Similarly, in the complexes with Dihycid and Ambenzyne, hydrogen bonds were crucial for stabilising the protein–ligand interactions. Residues such as GLU93, LEU175, THR382, and ASP148 in the Dihycid complex and CYS826, ASP827, ASP1190, and ASN1067 in the Ambenzyne complex formed hydrogen bonds with the respective ligands and water molecules, indicating specific recognition patterns and binding orientations. Pi-cation interactions were observed in several complexes, particularly with residues like HIS673, TYR400, LYS531, and ARG1111, where these aromatic residues engage with positively charged functional groups or benzene rings of the ligands. These interactions contribute to the complexes’ stability, influencing binding affinity and specificity. Pi–pi interactions involve stacking aromatic rings from the protein and ligand, as noted with residues such as HIS173, TYR331, TYR400, and TYR632. These interactions are important in molecular recognition, often providing additional stabilisation forces beyond hydrogen bonding. Salt bridges involving charged residues like ASP and GLU, with positively charged groups of the ligands (N+H3 atoms), were evident in complexes such as Ambenzyne and Amiflupipquamine. These electrostatic interactions contribute significantly to the binding affinity by forming solid and long-range interactions, stabilising the protein–ligand complex. Each ligand (Alparabinos, Dihycid, Ambenzyne, Amiflupipquamine, and Ametchomine) exhibits unique interactions with the protein PfSEA, suggesting distinct binding modes and specificity. For instance, Ametchomine forms hydrogen bonds predominantly with residues ASP1202, ASN1203, and LYS1090, while Ambenzyne engages in extensive interactions with CYS826, ASP827, and ASN1067. These observations indicate that the protein accommodates different ligands through varied molecular recognition patterns, adapting its binding site to complement the ligand’s chemical structure and functional groups. Understanding the detailed interactions between proteins and ligands is crucial for rational drug design and development. By elucidating the specific binding modes, such as hydrogen bonding networks, pi-cation interactions, and salt bridges, researchers can optimise ligand structures to enhance binding affinity and specificity. Moreover, insights into these interactions provide a foundation for structure-based drug design approaches, aiming to develop novel therapeutics that target PfSEA effectively.

### 2.7. MM\GBSA Studies

**Figure 9:**
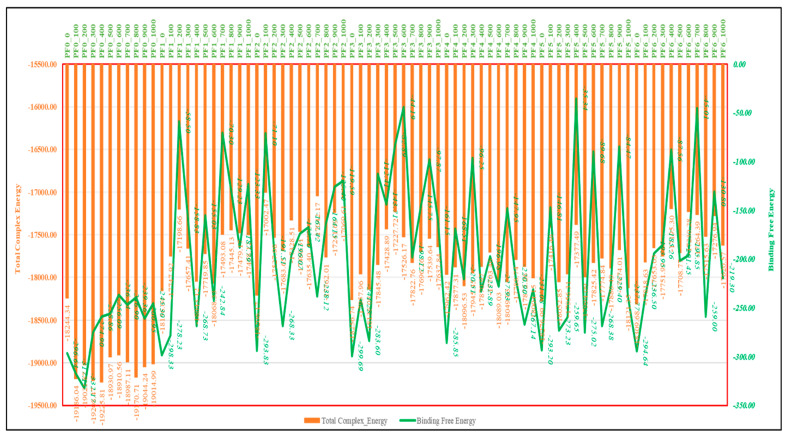
The MM/GBSA (Molecular Mechanics Generalized Born Surface Area) computations’ results, where we have shown the binding free energy in green and the total complex energy in orange for (**PF0**) PfSEA-1, as well as PfSEA-1 in complex with (**PF1**) Chlobenethyzenol, (**PF2**) Ametchomine, (**PF3**) Amiflupipquamine, (**PF4**) Ambenzyne, (**PF5**) Dihycid, and (**PF6**) Alparabinos.

The MMGBSA results for PfSEA-1 over 0 to 1000 frames (MD simulation) show fluctuations in total complex and binding free energy, reflecting the dynamic interactions during the molecular dynamics simulation. For instance, in the PF0 case (PfSEA-1 protein), the binding free energy starts at −296.64 kcal/mol and reaches a minimum of −332.17 kcal/mol at frame 200, indicating binding solid stability. However, the energy increases to −236.54 kcal/mol by frame 600, suggesting reduced binding affinity. In PF1 (PfSEA-1 in complex with Chlobenethyzenol), binding free energy starts at −298.33 kcal/mol, fluctuates significantly, and drops to a minimum of −58.50 kcal/mol at frame 200, indicating a considerable decrease in binding stability. By frame 600, the energy improves slightly to −242.84 kcal/mol, though it remains less stable than the initial values. For PF2 (PfSEA-1 in complex with Ametchomine), the binding free energy begins at −293.83 kcal/mol and drops significantly to −71.10 kcal/mol at frame 100 but then recovers to −191.51 kcal/mol by frame 200 (Figure 9). This pattern highlights significant instability at specific points, although some frames demonstrate moderate binding affinity. In PF3 (PfSEA-1 in complex with Amiflupipquamine), the binding free energy fluctuates from −299.69 kcal/mol at the start to −283.60 kcal/mol by frame 200, reflecting a relatively stable binding interaction. However, the energy increases to −44.19 kcal/mol at frame 600, indicating a marked reduction in binding stability. The higher free energy of Amiflupipquamine, despite having fewer hydrogen bonds and salt bridges than Alparabinos, can be attributed to multiple factors. Strong van der Waals and hydrophobic interactions may contribute significantly to its binding, compensating for the lack of hydrogen bonds. Additionally, entropic effects play a role; if Amiflupipquamine undergoes less conformational restriction upon binding, it retains more favourable entropy, increasing its total free energy. Solvation effects may also be a factor; if Amiflupipquamine is highly hydrophilic in solution, it may require more energy for desolvation before binding, leading to a higher free energy cost. Furthermore, electrostatic interactions, π-stacking, and conformational strain could influence its stability. If the ligand adopts an unfavourable bound conformation, more energy may be required to maintain it. In contrast, Alparabinos may have a better binding pocket fit with minimal strain, leading to lower overall free energy despite forming more hydrogen bonds. This highlights that while hydrogen bonding is important, hydrophobicity, entropy, and solvation effects can significantly impact ligand-binding energetics. The PF4 (PfSEA-1 in complex with Ambenzyne) series starts at −285.85 kcal/mol, sees a decrease to −168.51 kcal/mol by frame 100, and fluctuates with significant drops to −96.25 kcal/mol at frame 300. The energy fluctuates again, reaching −232.89 kcal/mol at frame 400, suggesting varying binding interactions. The PF5 (PfSEA-1 in complex with Dihycid) results show an initial binding free energy of −293.20 kcal/mol, sharply increasing to −35.34 kcal/mol by frame 400, reflecting significant instability. However, the binding energy improves to −237.77 kcal/mol by frame 1000. Lastly, PF6 (PfSEA-1 in complex with Alparabinos) starts at −294.64 kcal/mol, drops to −87.56 kcal/mol by frame 400, and fluctuates to −219.30 kcal/mol by frame 1000 (Figure 9). These fluctuations indicate varying degrees of binding stability throughout the simulation. These energy fluctuations impact the stability and efficacy of ligand binding to PfSEA-1, with specific frames showing stronger interactions, guiding potential optimisation in drug development. PF3 appears to be the best complex among PF1 and PF6. It maintains relatively vital binding energies, particularly around −283.60 kcal/mol (frame 200), and although it fluctuates, it shows significant binding stability compared to the other complexes (Figure 9).

## 3. Discussion

Our study involved crucial steps, from modelling to identifying and validating potential compounds. The 50-nanosecond MD simulation of the PfSEA-1 protein revealed substantial stabilisation and structural optimisation. The significant decrease in total and potential energy from 11,200 kcal/mol to −55,700 kcal/mol underscores a profound stabilisation of the protein structure. This stabilisation is evidenced by the detailed analysis of various energy components, which reflect the protein’s adaptation to a more energetically favourable conformation. Notably, the decrease in angle bending energy from 2960 kcal/mol to 1550 kcal/mol and torsion angle energy from 2120 kcal/mol to 885 kcal/mol suggests a relaxation and optimisation of bond angles and torsional conformations. This improved stability is further supported by the decrease in 1,4 Lennard-Jones energy, 1,4 electrostatic energy, Lennard-Jones energy, and electrostatic energy. These changes indicate enhanced non-bonded interactions, better electrostatic stabilisation, and a more favourable interaction landscape. The observed flexibility in several residues during the simulation highlights regions of pronounced mobility and potential structural adaptability. Significant fluctuations in the N-terminal and C-terminal regions, internal residues, and secondary structures suggest dynamic interactions or structural adaptations. These fluctuations are critical for understanding the protein’s functional mechanisms, as they provide insights into how the protein may adapt its structure to perform specific biological tasks or interact with other molecules. The dynamic behaviour of these residues underscores their importance in maintaining the protein’s flexibility and adapting to its functional requirements in biological contexts. The molecular docking studies provided comprehensive insights into the binding affinities and interaction specifics between the protein PfSEA and six ligands. The PfSEA–Chlobenethyzenol complex demonstrated the strongest binding affinity with a docking score of −8.107 kcal/mol. The formation of hydrogen bonds, salt bridges, and halogen bonds plays a crucial role in stabilising these protein–ligand complexes. The detailed interaction profiles for each ligand highlight the differential binding affinities and interaction strengths with the PfSEA protein, indicating their potential as therapeutic agents. The varying MMGBSA binding energies, particularly the significant electrostatic contributions observed for the PfSEA–Chlobenethyzenol complex, underscore the importance of electrostatic interactions in ligand binding. The ligand efficiency and ligand efficiency sa values further emphasise the favourable binding characteristics of these complexes. The detailed analysis of these interactions provides valuable insights into the potential efficacy of these ligands as therapeutic agents targeting the PfSEA protein. The molecular interaction fingerprint (MIF) analysis revealed fundamental interactions for stabilising the docked PfSEA-1 protein–ligand complexes with the six ligands. Identifying specific residues involved in these interactions provides a deeper understanding of the molecular basis of ligand binding and stabilisation. The frequency and distribution of these residues, particularly those involved in hydrogen bonding, salt bridges, and hydrophobic interactions, highlight these interactions’ critical role in maintaining the protein–ligand complexes’ stability and efficacy.

The DFT-based quantum mechanical optimisation of the six drug candidates provided critical insights into their electronic and vibrational characteristics, solvation effects, and potential efficacy as therapeutic agents. The significant differences in gas and solution phase energies emphasise the importance of solvation in molecular stability. Alparabinos, with the highest solvation energy, demonstrates enhanced potential bioavailability and stability in aqueous environments. Conversely, Chlobenethyzenol, with minimal solvation energy, may experience reduced solubility and overall effectiveness. The HOMO–LUMO energy gaps provide insights into these drug candidates’ electronic properties and reactivity. The smaller HOMO–LUMO gap in Chlobenethyzenol implies higher reactivity, making it a potentially more effective compound, albeit possibly less stable. The vibrational frequency analysis further elucidates these molecules’ dynamic behaviour and stability. Negative frequencies, particularly in Alparabinos, indicate potential instability or significant conformational flexibility, which could impact their interaction with biological targets. The thermodynamic properties, such as zero-point energy, entropy, enthalpy, and free energy, provide further insights into these drug candidates’ stability and potential efficacy. The detailed analysis of these properties underscores the importance of computational studies in the rational design and optimisation of new pharmaceuticals. The comprehensive analysis of Alparabinos and Chlobenethyzenol, in particular, highlights their unique characteristics that could influence their bioavailability and effectiveness, making them promising candidates for further drug development.

The molecular dynamics simulation of the PfSEA-1 protein in a complex with the six ligands provided valuable insights into the stability and interactions of these complexes. The root-mean-square deviation (RMSD) and root-mean-square fluctuation (RMSF) analyses revealed significant deviations and fluctuations in the protein and ligand structures. The initial instability followed by stabilisation in some complexes, such as the PfSEA-1 and Alparabinos complex, indicates dynamic structural adaptations and interactions. Compared to other complexes, the significant deviations observed in the PfSEA-1 and Chlobenethyzenol complexes suggest less stability and potential challenges in maintaining a stable interaction. The detailed analysis of fluctuating residues and interacting residues in each complex provides a deeper understanding of the molecular basis of these interactions and their impact on the stability and efficacy of the protein–ligand complexes. The comprehensive analysis of the protein structure preparation, molecular docking studies, molecular interaction fingerprints, DFT-based compound optimisation, and molecular dynamics simulation provides valuable insights into the stability, interactions, and potential efficacy of the PfSEA-1 protein and its complexes with six ligands. The significant stabilisation observed in the MD simulation, combined with the ligands’ detailed interaction profiles and electronic properties, highlights their potential as therapeutic agents targeting the PfSEA-1 protein. The insights gained from these studies underscore the importance of detailed computational and experimental analyses in the rational design and optimisation of new pharmaceuticals for therapeutic applications.

Chlobenethyzenol, despite its strong binding affinity and stable interactions with PfSEA-1, exhibits potential solubility concerns that may affect its bioavailability. Computational predictions suggest moderate aqueous solubility, which could impact its systemic absorption and therapeutic efficacy. Strategies such as prodrug development, salt formulation, or nanoparticle-based delivery systems could be explored to enhance solubility while maintaining stability. Given the favourable RMSD/RMSF results indicating minimal structural fluctuations and strong protein–ligand interactions, optimising solubility could further improve its drug-like properties. This study highlights PfSEA-1 as a promising target for novel antimalarial drug development. The identified drug candidates demonstrated strong binding affinities, structural stability, and favourable pharmacokinetic properties, making them viable contenders for therapeutic intervention. Given the increasing resistance to existing malaria treatments, targeting PfSEA-1 offers a novel mechanism of action that could help circumvent current drug resistance challenges. Integrating molecular docking, MD simulations, DFT analysis, and binding free energy calculations provides a comprehensive computational framework for rational drug discovery. These findings contribute to advancing targeted malaria therapies, offering new avenues for drug repurposing and design. While these computational predictions are promising, experimental validation through in vitro and in vivo studies is crucial to confirm efficacy, optimise pharmacokinetics, and assess potential toxicity. Future research should focus on preclinical evaluations and formulation strategies to enhance drug solubility and bioavailability. Ultimately, this work lays the foundation for developing next-generation antimalarial drugs, addressing a critical gap in malaria treatment strategies and supporting global eradication efforts.

## 4. Methods

The complete methodology is complex, and we plotted Figure 1 as a flowchart to understand it better. The detailed methods are in this section.

### 4.1. PfSEA-1 Sequence Downloading, Modelling, and Preparation

Homology modelling is crucial before docking for PfSEA-1 as it does not have a 3D crystal structure because it predicts the protein’s 3D structure based on homologous proteins with known structures [18]. Accurate structural models are essential for understanding the binding sites and interactions, enabling precise docking simulations. This facilitates the identification of potential inhibitors that can disrupt PfSEA-1 function, aiding in malaria treatment development [19]. We downloaded the sequence of PfSEA protein from the UniProt with Accession No A0A143ZXM2 (https://www.uniprot.org/, accessed on 1 July 2024) [20], which is 2074 amino acids long with a molecular weight of approximately 244,902 Daltons and plays a key role in the egress of merozoites from red blood cells, crucial for malaria parasite replication. We searched, using BLAST (https://blast.ncbi.nlm.nih.gov/, accessed on 1 July 2024) [21], for a template structure and identified PDBID: 3I2H [22] (https://rcsb.org/, accessed on 1 July 2024) as a template with the most similarity and downloaded it to model PfSEA-1 with Homology Modelling with Multiple Sequences Viewer/Editor [15,23,24,25]. The modelled structure was then prepared with the Protein Preparation Workflow (PPW) to achieve the optimised structure. We capped the termini, replaced hydrogens, created disulphide bonds and set zero bond orders for metals, optimised the structure with PROPKA, and minimised it with OPLS4 [15,26,27,28,29,30].

### 4.2. Molecular Dynamics Simulation and Validation

Molecular dynamics (MD) simulation is crucial for understanding PfSEA-1 because it provides detailed insights into the protein’s dynamic behaviour and structural stability under physiological conditions. We used the Desmond package, version (2024-2) (https://www.deshawresearch.com/resources.html) to simulate the modelled structure, where we built the system with the System Builder tool in Maestro [15,31]. We selected the TIP3P water model with 10 × 10 × 10Å distances for the box, and no ions were added as the system was already neutral; this was followed by minimisation to fix the box on the protein, and we used the OPLS4 forcefield [15,27,32]. For the production run, we used the molecular dynamics panel, where we set a 50 ns simulation time, recording an interval of 50 ps with a total 1000 frames in an NPT ensemble, 300K temperature, and 1.01325 bar pressure, and relaxed the model before simulation [15,33,34,35,36]. The production run was completed, the structure was validated with the Ramachandran Plot, and the deviation and fluctuations were computed. Further, in the well-optimised structure, we removed the water and minimised it again with the PPW panel to obtain its lowest energy level for all the docking when compared to other studies [37,38].

### 4.3. Ligand Library Preparation

Ligand library collection and preparation involve gathering diverse small molecules (ligands) and optimising their structures for computational studies. This includes filtering for drug-like properties, minimising energy states, and ensuring correct protonation states. We downloaded the complete Drug Bank (https://go.drugbank.com/, accessed on 1 July 2024) database and prepared it with the LigPrep tool in Maestro [39,40]. We browsed the complete ligand library, set the filter to 500 atoms, and used the OPLS4 force field [15,27]. We set the Epik classic for ionisation and generated possible states at a target pH of 7 ± 2, and desalt and tautomer generation were set [15,37,41]. The stereoisomers’ computations were set to retain the specified chiralities, generating 32 per ligand at most and writing the outputs in SDF for future use [42].

### 4.4. Molecular Docking with Simulation-Minimised Structure

Glide grid generation involves creating a 3D grid around the target protein’s active site to define potential binding pockets for molecular docking. This grid guides the docking process by mapping regions where ligands can interact with the protein, ensuring accurate and efficient identification of potential inhibitors [15,43,44]. We used the Receptor Grid Generation Tool in Maestro (2024-2), where we generated the grid on complete protein by checking the centroid of selected residues, and we specified ‘all’ residues to generate the grid on complete protein and increased the size of the box to make it fit appropriately on the protein. The glide grid generated a zip file which contained the complete coordinates and structure of the protein for docking studies [15,43,44,45]. Molecular docking is a crucial technique for identifying potential drug candidates, and we used it against PfSEA-1 to predict how small molecules (ligands) bind to the protein’s active sites. This process involves simulating the interactions between the protein and a library of ligands to evaluate binding affinities and select compounds that effectively inhibit PfSEA-1 [46]. For the molecular docking, we used the Virtual Screening tool (VSW), where we browsed the ligand library and set the QikProp and Lipinski’s rule filter to remove the compounds that did not pass the criteria [15,16,17,47]. We left the preparation step, and in the receptor tab, we added the prepared grid’s zip file and proceeded to the docking. In the docking tab, we used Epik state penalties for docking and docking the ligands with High Throughput Virtual Screening (HTVS), Standard Precision (SP), and Extra Precise (XP) algorithms [15,37,41]. The top 10% of HTVS ligands were passed to be redocked with SP, the top 10% of SP were passed to be redocked with XP, and all 100% XP results computed with 4 poses were passed to pose-process with Prime MM\GBSA [48]. The results were further exported to analyse the top compounds with the highest (negatively) docking scores and bonds [15,43,44,45].

### 4.5. Interaction Fingerprinting and Pharmacokinetics of Identified Candidates

Interaction fingerprints of docked poses provide a detailed map of the interactions between a ligand and the target protein, including hydrogen bonds, hydrophobic contacts, and electrostatic interactions. These fingerprints help evaluate the strength and nature of binding, identify key residues involved in ligand binding, and guide the optimisation of drug candidates to improve efficacy and specificity. We used the Interaction Fingerprints tool in Maestro, selected all 5 protein–ligand complexes, checked with the receptor–ligand complexes option, went further with all contact types, generated the fingerprints, and analysed them in the matrix [15]. The main plot was coloured for N to C terminals, the non-interacting residues were removed, and the counts of residue and ligand interactions were plotted. The pharmacokinetics of drug candidates encompasses studying how a drug is absorbed, distributed, metabolised, and excreted in the body. Key parameters include bioavailability, half-life, volume of distribution, and clearance rates. Understanding these factors is crucial for predicting the drug’s behaviour in the human body, optimising dosage regimens, and ensuring safety and efficacy in treating diseases like malaria. For all of the pharmacokinetics computations, we used the QikProp tool and then compared the results with standard values among all 5 identified candidates [15,16,17,49].

### 4.6. Optimisation Studies with Density Functional Theory

Density functional theory (DFT) is a quantum mechanical modelling method used to investigate the electronic structure of molecules and condensed matter systems. DFT approximates the electronic density to efficiently determine the properties of many-electron systems. It is widely used in computational chemistry and materials science for its balance between accuracy and computational feasibility, aiding in predicting molecular behaviour, reaction mechanisms, and material properties. We used the Optimisation panel (Jaguar) in Maestro for the DFT computations, where we selected the B3LYP-D3 theory and 6-31G**, and the automatic SCF spin treatment for DFT was kept [15,50,51]. The SCF accuracy was set to a quick level, and atomic overlap was used for the initial guess. The convergence criteria were set to 48 iterations, energy change was set to 5e-05, and RMS density was set to 5 × 10^−6^. Further, in the optimisation, we set a maximum of 100 steps, switched to analytic integrals near convergence, used default criteria with the Schlegel guess for the initial Hessian, and computed the surface properties of molecular orbitals, density and potential. The electrostatic potential and average local ionisation energy were computed (Kcal/mol), along with noncovalent interactions at a 20 pts/Å density. The spin density and HOMO–LUMO were also computed. We used the PBF solvent model and water as a solvent for solvation and optimised them in the gas phase. A QM-convergence monitor was used to analyse the results [15].

### 4.7. Molecular Dynamics Simulation and MM\GBSA Computations

Molecular dynamics (MD) simulation is a computational technique that models the physical movements of atoms and molecules over time. By applying Newtonian mechanics, MD simulations provide detailed insights into the structural dynamics, conformational changes, and interactions of biomolecules under various conditions. This method is essential for understanding the behaviour of proteins like PfSEA-1, aiding in drug design by predicting how potential drug candidates interact with the target protein in a realistic, time-evolved environment. For MD simulation, we used the Desmond package (https://www.deshawresearch.com/resources.html), which completed the simulation in three phases: system setup, production run, and analysis of the results [15,31]. For the system setup, we used the System Builder tool in Maestro to select the TIP3P water model in an orthorhombic box with a buffer box size calculation method with 10 × 10 × 10 Å distances and minimised the volume [15,27,32,38]. The ion and salt placements were excluded within 20 Å and added 17Na^+^, 17 Na^+^, 18Na^+^, 21Na^+^, and 20Na^+^ in PfSEA in complex with Alparabinos, Dihycid, Ambenzyne, Ametchomine, Amiflupipquamine, and Chlobenethyzenol, respectively, and used the OPLS4 force field. For the MD simulation production run, we used the molecular dynamics panel, where we loaded the built system file and set 100 ns as the simulation time with no elapses and 100 ps for recording intervals with a 1.2 energy level that recorded 1000 frames. We used the NPT ensemble class at 300 K and a 1.01325 bar pressure and relaxed the system before MD simulation [15,36]. We used the simulation interaction diagram tool to analyse the deviation, fluctuation, and intermolecular interactions for the analysis. The trajectory files were saved for further use for the MM\GBSA (Molecular Mechanics Generalized Born Surface Area) computations, a computational method for estimating the free energy of binding biomolecular complexes, such as protein–ligand interactions. MM\GBSA provides a detailed and dynamic view of binding energetics over time when applied to MD simulation trajectories. This approach combines molecular mechanics calculations with implicit solvent models, balancing accuracy and computational efficiency. For each snapshot, the binding free energy is calculated using the MM\GBSA method that involves molecular mechanics energy computations, including bonded interactions (bonds, angles, and dihedrals) and non-bonded interactions (van der Waals and electrostatic forces), solvation energy that estimates the solvation free energy using the generalized born (GB) model for polar solvation and a surface area (SA) term for non-polar solvation, and the most crucial one, binding free energy, that combines these energies to estimate the binding free energy, typically as

ΔG_bind = ΔE_MM + ΔG_GB + ΔG_SA − TΔS

where ΔE_MM is the molecular mechanics energy, ΔG_GB is the polar solvation energy, ΔG_SA is the non-polar solvation energy, and TΔS is the entropic contribution (often estimated separately). We used this command to run the MM\GBSA on the MDS trajectory [15,52]:

export SCHRODINGER=/opt/Schrodinger-VERSION/

$SCHRODINGER/run thermal_mmgbsa.py desmond_md_job_NAME-out.cms

## 5. Conclusions

Based on the comprehensive analyses conducted on the PfSEA-1 protein and its interactions with various ligands, namely, Chlobenethyzenol, Ametchomine, Amiflupipquamine, Ambenzyne, Dihycid, and Alparabinos, several conclusions can be drawn regarding their suitability as potential drugs targeting PfSEA-1. Molecular dynamics simulations indicated that these ligands effectively stabilise the PfSEA-1 protein structure. Significant decreases in total potential energy, Lennard-Jones, and electrostatic energies post-simulation underscored their role in enhancing protein stability. Moreover, the analysis of root-mean-square deviation (RMSD) and root-mean-square fluctuation (RMSF) highlighted that Ametchomine and Amiflupipquamine complexes exhibited the lowest deviations, indicative of stable protein–ligand interactions over the 100-nanosecond simulation period. Conversely, complexes such as Chlobenethyzenol and Ambenzyne showed initial instability but stabilised over time, suggesting potential viability with further optimisation. Molecular interaction fingerprints (MIFs) identified critical residues involved in stabilising these complexes, which are crucial for their binding affinity and specificity. Hydrogen bonds, salt bridges, and pi-cation contacts were pivotal in forming stable interactions between the ligands and PfSEA-1, enhancing their potential as therapeutic agents. These findings collectively support the hypothesis that these compounds can effectively modulate PfSEA-1 activity, making them promising candidates for further drug development efforts targeting malaria; however, more experimental studies are needed for these results to be more conclusive.

## Figures and Tables

**Table 1:** Various energy components of the protein before and after simulation.

Energy Component	Before MDS (kcal/mol)	After MDS (kcal/mol)
Total Energy of the System	1.12 × 10^4^	−5.57 × 10^4^
Total Potential Energy	1.12 × 10^4^	−5.57 × 10^4^
Total Kinetic Energy	0	0
Temperature of the System	0 K	0 K
Bond Stretch Energy	5.37 × 10^2^	5.63 × 10^2^
Angle Bending Energy	2.96 × 10^3^	1.55 × 10^3^
Torsion Angle Energy	2.12 × 10^3^	8.85 × 10^2^
Restraining Energy for Torsions	0	0
1,4 Lennard-Jones Energy	3.68 × 10^3^	2.86 × 10^3^
1,4 Electrostatic Energy	8.69 × 10^2^	8.46 × 10^2^
Lennard-Jones Energy	−2.17 × 10^3^	−9.96 × 10^3^
Electrostatic Energy	−2.92 × 10^3^	−5.34 × 10^4^
H-bond Energy	0	0

**Table 2:** The docking scores among PfSEA-1 and ligands and other calculations during the multi-sample docking and MM\GBSA calculations.

S No	Drug Bank	Drug Name	Docking Score	MMGBSA dG Bind Coulomb	MMGBSA dG Bind Covalent	MMGBSA dG Bind Hbond
1	DB07521	Chlobenethyzenol	−8.107	−445.18	4.98	−1.95
2	DB08019	Ametchomine	−7.005	−319.81	4.06	−3.53
3	DB08750	Amiflupipquamine	−6.94	−385.43	1.88	−2.69
4	DB02044	Ambenzyne	−6.505	−375.08	0.98	−3.04
5	DB03946	Dihycid	−6.225	78.17	6.11	−3.87
6	DB03142	Alparabinos	−4.481	−40.44	8.54	−4.65
**S No**	**Drug Bank**	**Drug Name**	**Prime Hbond**	**Prime vdW**	**Ligand Efficiency**	**Ligand Efficiency sa**
1	DB07521	Chlobenethyzenol	−328.93	4.67284 × 10^20^	−0.262	−0.822
2	DB08019	Ametchomine	−330.51	4.67284 × 10^20^	−0.269	−0.798
3	DB08750	Amiflupipquamine	−329.67	4.67284 × 10^20^	−0.257	−0.771
4	DB02044	Ambenzyne	−330.02	4.67284 × 10^20^	−0.5	−1.177
5	DB03946	Dihycid	−330.85	4.67284 × 10^20^	−0.566	−1.258
6	DB03142	Alparabinos	−331.63	4.67284 × 10^20^	−0.448	−0.965

**Table 3:** The ADMET values of the identified ligands against the standard QikProp values.

Descriptors	Standard	Alparabinos	Dihycid	Ambenzyne	Amiflupipquamine	Ametchomine	Chlobenethyzenol
#stars	0–5	0	0	0	0	0	5
#amine	0–1	3	3	1	1	0	0
#amidine	0	0	0	0	1	0	0
#acid	0–1	0	0	0	0	1	0
#amide	0–1	0	0	0	0	0	0
#rotor	0–15	10	9	4	5	3	5
#rtvFG	0–2	1	0	0	0	0	1
CNS	−2 (inact), +2 (act)	1	0	−1	0	−2	−2
mol MW	130.0–725.0	484.486	373.928	367.425	177.249	154.122	150.131
dipole	1.0–12.5	3.471	3.578	5.54	4.115	5.953	1.757
SASA	300.0–1000.0	833.794	598.359	641.741	448.687	331.243	312.149
FOSA	0.0–750.0	406.592	283.638	175.215	157.785	0	127.898
FISA	7.0–330.0	88.112	114.794	163.364	145.123	206.617	184.251
PISA	0.0–450.0	167.583	151.102	268.468	145.779	124.626	0
WPSA	0.0–175.0	171.507	48.825	34.694	0	0	0
volume-1	500.0–2000.0	1480.343	1152.562	1138.44	710.665	505.322	484.044
donorHB	0.0–6.0	4	5	5	4	3	4
accptHB	2.0–20.0	8.2	6	6	2.5	3.5	8.5
dip^2/V	0.0–0.13	0.008138	0.011108	0.0269578	0.0238265	0.0701263	0.0063776
ACxDN^.5/SA	0.0–0.05	0.0196691	0.022422	0.0209063	0.0111436	0.0183013	0.0544611
glob	0.75–0.95	0.7533806	0.8884775	0.8216352	0.8583498	0.9262546	0.9551232
QPpolrz	13.0–70.0	48.269	35.636	39.402	20.468	13.333	10.007
QPlogPC16	4.0–18.0	16.213	12.378	12.739	7.576	6.013	5.298
QPlogPoct	8.0–35.0	26.522	22.398	24.217	13.742	11.442	13.225
QPlogPw	4.0–45.0	15.255	14.233	16.564	9.996	9.949	14.851
QPlogPo/w	−2.0–6.5	3.292	1.375	2.017	0.269	0.031	−1.745
QPlogS	−6.5–0.5	−3.545	0.263	−3.599	−0.885	−0.798	−0.705
CIQPlogS	−6.5–0.5	−3.161	−1.696	−3.845	−0.857	−1.429	−0.314
QPlogHERG	concern below −5	−8.5	−6.236	−6.425	−5.245	−1.512	−2.258
QPPCaco	<25 poor, >500 great	22.441	12.532	69.763	103.895	27.552	177.276
QPlogBB	−3.0–1.2	0.144	−0.032	−0.807	−0.653	−1.223	−1.084
QPPMDCK	<25 poor, >500 great	96.199	10.905	47.685	47.346	12.965	76.248
QPlogKp	−8.0–−1.0	−5.718	−6.364	−5.263	−7.326	−4.6	−4.435
IP(eV)	7.9–10.5	8.49	8.367	8.515	8.408	9.292	10.868
EA(eV)	−0.9–1.7	0.737	0.131	0.649	0.265	0.609	−2.286
#metab	1–8	5	6	3	4	2	3
QPlogKhsa	−1.5–1.5	0.411	0.025	0.131	−0.456	−0.9	−0.839
HumanOralAbs	N/A	2	2	3	2	2	2
% HumanOralAbs	>80% is high, <25% is poor	70.402	54.65	71.754	64.615	52.903	56.972
SAfluorine	0.0–100.0	0	0	34.694	0	0	0
SAamideO	0.0–35.0	0	0	0	0	0	0
PSA	7.0–200.0	74.425	78.123	99.369	73.945	93.526	97.786
#NandO	2–15	6	5	6	3	4	5
RuleOfFive	maximum is 4	0	0	0	0	0	0
RuleOfThree	maximum is 3	1	1	0	0	0	0
#ringatoms	N/A	21	17	21	6	6	5
#in34	N/A	0	0	0	0	0	0
#in56	N/A	21	17	21	6	6	5
#noncon	N/A	8	4	5	0	0	4
#nonHatm	N/A	31	26	27	13	11	10
Jm	N/A	0.000264	0.296407	0.000505	0.001091	0.616045	1.086211

## Data Availability

Data are contained within the article.
